# Determinants of MRSA carriage/infection in the Netherlands

**DOI:** 10.1186/1753-6561-5-S6-O82

**Published:** 2011-06-29

**Authors:** M van Rijen, J Kluytmans, the CAM-study group

**Affiliations:** 1 Laboratory for Microbiology and Infection Control, AMPHIA HOSPITAL, Breda, Netherlands

## Introduction / objectives

Recently, the epidemiology of MRSA has changed. Community-acquired MRSA is increasing. The objective of this study was to investigate the determinants of MRSA carriage/infections in The Netherlands.

## Methods

All newly identified patients with MRSA in seventeen hospitals dispersed over the Netherlands were included from January 2009 until December 2010. MRSA determinant analysis was done based on the patient’s history combined with molecular typing results and then classified in risk groups described in the national infection prevention guidelines.

## Results

In two years 1021 patients (369 inpatients and 652 outpatients) were found to be MRSA positive for the first time. Analysis of risk factors revealed that 59.2% (n=604) had been exposed to pigs/veal calves, 7.3% (n=75) had been admitted to a foreign hospital, 4.3% (n=44) were considered to be caused by nosocomial transmission, 0.5% (n=5) were colonised due to transmission in a nursing home, 1.8% (n=18) were adoption children , 0.2% (n=2) were dialysis patients from a foreign country and 26.5% (n=271) could not be classified in a known risk group. The patient’s history was not available in 0.2% (n=2).

Panton-Valentine Leucocidin toxic gene expression was mainly seen in the group with unknown risk factor (57 positive, 209 negative, 5 unknown) and only sporadically in other groups (7 positive, 737 negative, 6 unknown, *p*<*0.001*). Remarkably, 21.7% of the strains in the group with unknown determinants belonged to the clonal complex ST-398 (59/271).

## Conclusion

The majority (n=604) of newly identified MRSA carriers were related to exposure to live-stock. From all other cases (n=415), the majority (n=271) reported no known source. Strains with spa-types indicative for Livestock Associated MRSA were found in 16.5% of individuals who did not report contact to livestock. This indicates that LA-MRSA is spreading in the community.

## Disclosure of interest

None declared.

